# Oligodendrocytes in intracerebral hemorrhage

**DOI:** 10.1111/cns.13193

**Published:** 2019-08-14

**Authors:** Minkyung Kang, Yao Yao

**Affiliations:** ^1^ Department of Pharmaceutical and Biomedical Sciences University of Georgia Athens GA USA

**Keywords:** intracerebral hemorrhage, myelination, oligodendrocyte precursor cells, oligodendrocytes

## Abstract

Intracerebral hemorrhage (ICH) is a cerebrovascular disorder with high mortality and disability rates. Although a lot of effort has been put in ICH, there is still no effective treatment for this devastating disease. Recent studies suggest that oligodendrocytes play an important role in brain repair after ICH and thus may be targeted for the therapies of ICH. Here in this review, we first introduce the origin, migration, proliferation, differentiation, and myelination of oligodendrocytes under physiological condition. Second, recent findings on how ICH affects oligodendrocyte biology and function are reviewed. Third, potential crosstalk between oligodendrocytes and other cells in the brain is also summarized. Last, we discuss the therapeutic potential of oligodendrocyte‐based treatments in ICH. Our goal is to provide a comprehensive review on the biology and function of oligodendrocytes under both physiological and ICH conditions.

## 1 INTRODUCTION

Stroke is the 5th leading cause of death and the leading cause of long‐term disability in the United States.^1^, ^2^ Based on the pathology, stroke is categorized into two types: ischemic stroke that occurs when blood supply to the brain is impeded, and hemorrhagic stroke that occurs when blood vessel ruptures in or around the brain. Depending on the site of bleeding, hemorrhagic stroke can be further divided into intracerebral hemorrhage (ICH) and subarachnoid hemorrhage (SAH).

ICH, which accounts for 10%‐15% of all stroke subtypes,^3^ is the most common type of hemorrhagic stroke. Its major risk factors include aging and hypertension, which are usually difficult to control. When ICH occurs, blood leaks into brain parenchyma resulting in primary and secondary brain damage. The former is predominantly caused by mass effect and tissue disruption. The latter is mainly due to inflammatory reaction and erythrocyte lysis. Although there is not much we can do about the primary damage, a lot of effort has been made to attenuate secondary damage. Unfortunately, there is still no effective treatment for this devastating disorder. Recent studies suggest that a better understanding of remyelination and white matter injury may shed new light on the treatment of ICH.^4^ The cell type that actively regulates remyelination and white matter injury is oligodendrocytes.^5^, ^6^ Here in this review, we first introduce the origin, migration, proliferation, differentiation, and myelination of oligodendrocytes under physiological condition. Second, recent findings on how ICH affects oligodendrocyte biology and function are reviewed. Third, potential crosstalk between oligodendrocytes and other cells in the brain is summarized. Last, we discuss the therapeutic potential of oligodendrocyte‐based treatments in ICH. The search criteria include a combination of “intracerebral hemorrhage” or “hemorrhagic stroke” and oligodendrocyte‐related keywords, such as “oligodendrocyte,” “OPC,” “OPC proliferation,” “oligodendrocyte differentiation,” “oligodendrocyte myelination,” “oligodendrocyte development,” “oligodendrocyte heterogeneity,” “oligodendrocyte morphology,” and “oligodendrocyte function.”

## 2 OLIGODENDROCYTE PHYSIOLOGY

### 2.1 Oligodendrocyte Origin

In the brain, oligodendrocytes are derived from oligodendrocyte progenitor cells (OPCs), which originate from neuroepithelial progenitor cells (NPCs) of neuroepithelium in the embryonic neural tube and forebrain.^7^, ^8^ In mice, NPCs become radial glial cells at about embryonic day (E) 9, which differentiate into OPCs at a later time.^7^, ^8^, ^9^

A study by Kessaris et al. Elegantly demonstrated three waves of OPC generation in the forebrain.^10^ The first wave arises at the medial ganglionic eminence (MGE) and anterior entopeduncular area (AEP) in the ventral forebrain at E11.5‐E12.5.^10^ These OPCs migrate across the telencephalon from ventral to dorsal area and account for most part of the embryonic forebrain until the next wave emanates. OPCs originated from the first wave, however, show significantly decreased number in most parts of the adult forebrain. The second wave emanates from the lateral and/or caudal ganglionic eminences (LGE and/or CGE) at around E15.^10^ These cells populate the cortical intermediate zone in embryonic brain and comprise most of the postnatal telencephalon. The third wave originates from the postnatal cortex at around the day of birth.^10^ OPCs generated from this wave are found only at dorsal telencephalon and mostly remain in the cortex until adulthood.

Interestingly, functional redundancy exists among different OPC populations. For example, ablation of one population leads to the substitution of the excised population by another population and normal survival and behavior of the resulting mice.^10^ In addition, it has been shown that OPCs from the diencephalon repopulate the telencephalon when all telencephalic OPCs are removed from the origins.^11^, ^12^ These findings suggest great plasticity of OPCs during development.^12^

### 2.2 Oligodendrocyte migration

Once OPCs are specified, multiple signaling cues guide them to their destination in the brain. It has been reported that the direction of OPC migration is largely determined by spatial gradients of BMPs (bone morphogenic proteins), Shh (sonic hedgehog), and Wnt proteins.^13^ In addition, growth factors, such as PDGF (platelet‐derived growth factor),^14^ VEGF (vascular endothelial growth factor),^15^ and FGF (fibroblast growth factor),^16^ are known to augment OPC migration. Furthermore, there is also evidence suggesting that brain vascularization regulates OPC migration.^17^ It has been shown that OPCs migrate (crawl along and jump between blood vessels) by physically contacting blood vessels in the brain.^17^ It should be noted that the exact molecular mechanisms underlying OPC migration remain largely unclear and need further investigation.

### 2.3 Oligodendrocyte proliferation

Unlike neurons, which have very limited capacity of proliferation, OPCs remain highly proliferative in adult brain.^18^ As the major population of proliferating cells in the adult central nervous system (CNS), OPCs maintain their density and number until later in life.^19^ Their ability of self‐renewal is closely related to stimulation of cell cycle and inhibition of differentiation. Mitogens and growth factors play critical roles in regulating cell cycle. For instance, PDGF, by binding to its receptor PDGFRα, enhances OPC proliferation and survival in vivo.^20^, ^21^ Similarly, FGF2, BDNF (brain‐derived neurotrophic factor), and NT‐3 (neurotrophin‐3) have also been shown to enhance OPC proliferation in vitro.^22^, ^23^, ^24^ Additionally, translocation of transcription factor Id2 (inhibitor of DNA binding 2) into nucleus has been demonstrated to induce OPC proliferation and inhibit their differentiation.^25^ Initiation of differentiation, on the contrary, slows down cell cycle and reduces OPC proliferation.^18^

### 2.4 OPC differentiation

Molecules that inhibit OPC proliferation usually act as inducers of OPC differentiation. For example, by inhibiting PDGF‐driven OPC proliferation, TGF‐β (transforming growth factor‐β) functions as a possible inducer of OPC differentiation.^26^ Similarly, translocation of Id2 from the nucleus to the cytoplasm precedes OPC differentiation.^25^ In addition, thyroid hormones have been shown to promote OPC differentiation.^27^, ^28^ Recently, there is evidence showing that microRNA also regulates OPC differentiation.^29^

**Figure 1:**
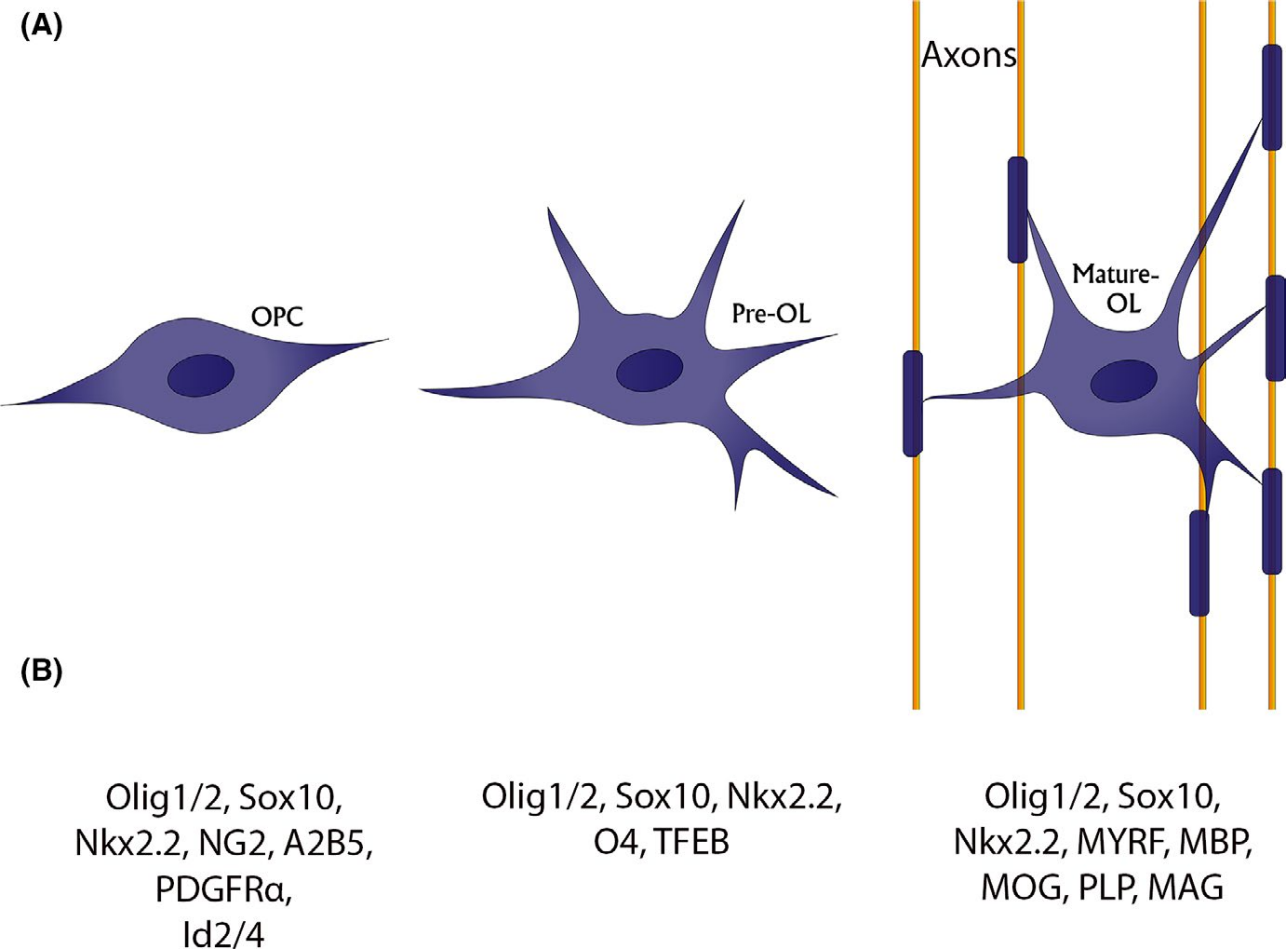
Diagram of oligodendrocyte differentiation. Morphological (A) and biochemical (B) features that characterize each differentiation stage

OPC differentiation involves two continuous steps: the differentiation of OPCs into immature postmitotic pre‐oligodendrocytes (pre‐OLs) and subsequent maturation of these pre‐OLs into myelinating oligodendrocytes (mature‐OLs).^13^, ^18^ This differentiation process involves striking changes at both morphological and biochemical levels (Figure 1). Structurally, the highly proliferative OPCs usually take a bipolar or oval shape.^30^, ^31^ Compared to OPCs, pre‐OLs have various processes, the number of which correlates with the extent of differentiation.^31^ Unlike pre‐OLs, mature‐OLs develop processes that enwrap neuronal axons, forming myelin sheaths.^31^

Biochemically, oligodendrocyte‐lineage cells express a variety of molecular markers at distinct differentiation stages. Upon lineage specification, Olig1/2, Sox10 (SRY‐Box transcription factor 10), and Nkx2.2 are substantially upregulated and persist throughout the life of oligodendrocyte‐lineage cells.^7^, ^13^, ^31^, ^32^, ^33^, ^34^, ^35^, ^36^, ^37^, ^38^, ^39^ Thus, these molecules are used as markers for oligodendrocyte‐lineage cells. Classical OPC markers include NG2 (neuron‐glial antigen 2), PDGFRα, and Id2/4.^34^, ^40^, ^41^, ^42^, ^43^, ^44^, ^45^ It should be noted that these markers are not OPC‐specific. For example, NG2 is also expressed in mural cells and PDGFRα is also found in fibroblasts.^46^, ^47^ Therefore, multiple markers should be used to identify OPCs. Markers for pre‐OLs are relatively less well characterized. A recent study reported that TFEB (transcription factor EB) is highly enriched in pre‐OLs and can be used as a pre‐OL marker.^48^ In addition, O4 has been also suggested as a pre‐OL marker,^40^, ^44^, ^49^ although there is also evidence showing that it is also expressed in mature‐OLs.^50^, ^51^ Mature‐OLs form myelin sheaths and express a group of unique proteins, including MYRF (myelin regulatory factor),^52^ MBP (myelin basic protein),^38^, ^44^, ^53^ MOG (myelin oligodendrocyte glycoprotein),^54^, ^55^, ^56^ PLP (proteolipid protein),^44^, ^53^, ^57^ and MAG (myelin‐associated glycoprotein).^58^, ^59^ These proteins are widely used as molecular markers for mature‐OLs. For a summary of the expression of these markers in oligodendrocyte‐lineage cells, please refer to Table 1.

**Table 1:** Markers of oligodendrocyte‐lineage cells

Markers	Stages		
	OPCs	Pre‐OLs	Mature‐OLs
Olig1/2	+	+	+
Sox10	+	+	+
Nkx2.2	+	+	+
NG2	+		
PDGFRa	+		
Id2/4	+		
TFEB		+	
O4		+	
MYRF			+
MBP			+
MOG			+
PLP			+
MAG			+

While OPCs and mature‐OLs are extensively studied, pre‐OLs are in general understudied. For example, fewer molecular markers are available for pre‐OLs compared to OPCs and mature‐OLs. The functions of pre‐OLs in physiological and pathological conditions remain largely unknown. Therefore, future studies should focus on elucidating the markers and functions of pre‐OLs.

### 2.5 Myelination

The major function of oligodendrocytes in the CNS is to myelinate axons. Myelinated axons contain myelin sheaths and gaps known as nodes of Ranvier. This unique structure allows faster action potential propagation via saltatory conduction,^60^ in which current flows and jumps from one node of Ranvier to the next. Without myelin sheaths, action potentials show lower amplitudes, longer latencies, decreased conduction velocity, and gradual dispersion,^61^ suggesting a crucial role of myelination in the propagation of electrical signal along axons. Consistent with this important function, myelination has been found to be indispensable for various neurological functions, including motor learning^62^, ^63^ and social behavior.^64^

Myelination is tightly coupled with OPC differentiation and is orchestrated by diverse cellular mechanisms. Upon differentiation, OPCs develop processes to wrap neuronal axons. Once a process first contacts an axon, that contact can be stabilized, creating an axoglial communication.^65^ Next, the myelin membrane establishes polarized domains after molecular translocation in the cell.^66^ This polarized tip of myelin then expands on the axon both laterally toward the nodes of Ranvier and radially via growing underneath the previously formed membrane.^67^ After lateral and radial expansion of myelin sheath follows myelin compaction, which is composed of intracellular and extracellular leaflet compaction.^68^ The intracellular compaction is done by neutralization of negatively charged myelin membrane.^65^ In this process, MBP neutralizes the membrane and pulls the inner leaflets of myelin sheaths close to each other, forming the major dense line (MDL).^69^, ^70^ The extracellular compaction, on the other hand, is a process where the outer surfaces of myelin membranes are brought together, creating intraperiod line (IPL).^71^ Compared to intracellular compaction, extracellular compaction is relatively less well understood.

Since myelin sheaths are in direct contact with axons, it has been speculated that axons play an important role in oligodendrocyte maturation and myelination. Various studies support this hypothesis. For instance, upon neuronal stimulation or activation in the brain, the thickness^72^ and length^73^ of myelin sheath as well as the number of myelin sheath generated by an oligodendrocyte^74^ are increased. In sharp contrast to these findings, however, there is also evidence showing that the presence of axons is unnecessary for myelination. It has been shown that oligodendrocytes are able to myelinate electron‐spun nanofibers in a neuron‐free culture system,^75^ suggesting that oligodendrocytes have an intrinsic propensity to wrap axons independent of neuronal activity. These results suggest that neuronal activity is not absolutely required for myelination, but it can enhance the extent of myelination. In addition, a recent study reported different myelination patterns toward distinct populations of axons from different brain regions,^76^ suggesting functional heterogeneity of oligodendrocytes. Consistent with this hypothesis, 13 distinct oligodendrocyte populations have been identified based on single‐cell RNA sequencing.^62^ What causes oligodendrocyte heterogeneity and how oligodendrocyte heterogeneity affects myelination remain unknown. These important questions need future investigation.

### 2.6 Signaling pathways regulating myelination

Myelination is a delicate process that requires tight regulation. Multiple signaling pathways have been reported to be involved in myelination. First, extracellular cellular matrix (ECM) molecules have been shown to actively regulate the myelination process in the CNS. For example, by binding to integrins, laminin and fibronectin lengthen the OPC processes.^77^, ^78^, ^79^ In addition, loss of laminin‐α2β1γ1 leads to reduced mature‐OLs and dysmyelinated axons in multiple brain regions.^80^ Furthermore, laminin‐α2β1γ1, by binding to integrin β1, has also been shown to regulate the thickness of myelin sheath.^81^, ^82^ Apart from integrins, dystroglycan can also mediate laminin's effect on myelination.^83^ It has been shown that dystroglycan function‐blocking antibody significantly decreases the complexity of myelin morphology.^83^ These results suggest a crucial role of ECM‐integrin/dystroglycan signaling pathway in myelination.

Next, there is also evidence showing that Fyn is a promyelinating factor. It has been shown that upregulation of Fyn in OPCs promotes the production of highly branched processes in vitro*.* 79 Consistent with this finding, the tyrosine kinase activity of Fyn, which is activated at the early stage of OPC differentiation, is required for the branching of processes and formation of myelin sheaths.^84^ In addition, the regulatory mechanism of Fyn on cytoskeletal rearrangement also contributes to myelination.^84^ For example, Fyn‐Tau‐tubulin cascade has been shown to be important for the outgrowth of oligodendrocyte processes.^85^, ^86^ Less and shorter oligodendrocyte processes are observed when the interaction of Fyn and Tau is inhibited.^86^ Together, these results suggest a crucial role of Fyn signaling in myelination.

In addition, many other signaling pathways have also been reported to participate in the regulation of myelination. First, mice lacking ERK1/2 (extracellular signal‐regulated protein kinases 1/2) in oligodendrocyte‐lineage cells fail to produce myelin sheaths with proper thickness and show a reduced expression of major myelin genes, including PLP and MBP,^87^ suggesting a promyelinating effect of ERK/MAPK signaling pathway. Next, overexpression of active AKT in oligodendrocytes leads to hypermyelination without affecting the survival of oligodendrocytes or OPCs,^88^ while mTOR (mammalian target of rapamycin) inhibitor rapamycin inhibits hypermyelination,^89^ suggesting that AKT/mTOR signaling pathway positively regulates myelination. Interestingly, although the presence of axons is not required for myelination,^75^ neuronal expression of type III neuregulin‐1 promotes myelination and positively regulates myelin thickness in the forebrain,^90^ indicating a critical role of neuronal signal in myelination. Additionally, there is also evidence supporting that Wnt^91^ and BDNF^92^ promote myelination. For a summary of the factors that affect oligodendrocyte biology, please refer to Table 2.

**Table 2:** Factors that affect oligodendrocyte biology

Migration	Proliferation	Differentiation	Maturation (Myelination)
BMP Shh Wnt PDGF VEGF FGF Brain vasculature	PDGF FGF2 BDNF NT‐3 Id2 (nucleus)	TGF‐β Id2 (cytoplasm) Thyroid hormones Micro RNA	Neuronal activity Type III neuregulin‐1 Integrin Dystroglycan Fyn ERK/MAPK AKT/mTOR Wnt BDNF

### 2.7 Age‐ and sex‐related differences in ICH and oligodendrocyte biology

Accumulating evidence suggests that age is a major risk factor for ICH.^93^, ^94^ It has been shown that aging increases the incidence of ICH due to worse chronic conditions, such as hypertension, atrial fibrillation, and diabetes.^3^ In addition, age‐related changes in brain vasculature, including increased blood‐brain barrier permeability, endothelial dysfunction, and decreased vascular density, also contribute to the pathogenesis of ICH.^3^ Apart from age, sex is another risk factor for ICH. It has been reported that ICH occurs more frequently in males (52.4%) than in females (47.6%) based on a study conducted on 515 acute primary ICH patients.^95^ The study also showed that women were older than men, although hematoma volume was similar in both sexes,^95^ suggesting that males are generally more susceptible to ICH than females.^95^ Similarly, a higher incidence (53.5%) of ICH was found in males in another study containing 2212 patients.^96^ Interestingly, this study identified male sex as a risk factor for hematoma expansion, which was defined as hematoma growth >33% or >6 mL from baseline ICH volume after 24 hours of onset.^96^

Whether age‐ and sex‐related differences exist in oligodendrocyte biology remains largely unknown. A recent study reported that OPCs have distinct molecular signatures at different ages.^97^ Specifically, OPCs display migrating molecular signature at E16 and proliferating molecular signature at P12.^97^ In addition, molecular signatures of both proliferation and differentiation decrease from P12 to P80.^97^ In another study, it has been demonstrated that the number of OPCs in both dorsal and ventral Ammon's horns decreases with age and that the number of OLs reduces in the ventral but not dorsal Ammon's horn with age,^98^ indicating region‐specific age‐related differences in the biology of oligodendrocyte‐lineage cells. Similarly, there is evidence supporting that sex may affect OL biology. For example, it has been shown that: (a) In young (6‐8 months) rats, males have significantly larger volumes of white matter, myelinated nerve fibers, and myelin sheaths than females^99^; and (b) in middle‐aged (18 months) rats, these volumes are much larger in females than males.^99^ In addition, OPC proliferation and maturation are regulated by sex hormones.^100^ First, OPCs isolated from female pups generated significantly more OLs than those isolated from male ones.^100^ Second, 17β‐estradiol, the major female sex hormone, prevented OPCs from exiting cell cycle in response to mitogen withdrawal, whereas progesterone and testosterone failed to do so.^100^ Third, progesterone‐treated cells demonstrated more complex (matured) morphology, while 17β‐estradiol‐ and testosterone‐treated cells showed less complex morphology.^100^ Further studies are needed to uncover the mechanisms underlying age‐ and sex‐related differences in oligodendrocyte biology.

## 3 EFFECTS OF ICH ON OLIGODENDROCYTE‐LINEAGE CELLS

### 3.1 Oligodendrocyte death after ICH

When ICH occurs, blood leaks into brain parenchyma, causing a series of changes, including iron toxicity and cell death.^101^, ^102^, ^103^ As the cell type that contains a high level of iron in the CNS, oligodendrocytes are very sensitive to iron overload^104^ and thus particularly susceptible to ICH injury. It has been reported that ICH induces oligodendrocyte death and demyelination in white matter,^105^, ^106^ where functional and morphological maintenance is highly dependent on oligodendrocytes and their myelin sheaths.^107^

How do oligodendrocytes die after ICH? On the one hand, there is evidence suggesting that apoptosis is responsible for oligodendrocyte death after ICH. For example, it was reported that oligodendrocytes expressed a significantly higher level of caspase‐3 at the injury site after internal capsule hemorrhage, compared to noninjured controls.^105^ Further mechanistic study revealed that ER stress and mitochondrial dysfunction contributed to oligodendrocyte apoptosis.^105^ Like in ICH, oligodendrocyte apoptosis also occurs in ischemia.^108^ Necrosis, on the other hand, has also been proposed to contribute to oligodendrocyte death after ICH, especially at the acute phase. For instance, it has been demonstrated that more than half of injured cells are necrotic 48 hours after injury in the collagenase‐induced ICH model.^109^

### 3.2 OPC proliferation after ICH

Based on that OPCs remain proliferative throughout life, it has been speculated that OPCs contribute to cell repopulation in the brain after injury.^19^ Consistent with this hypothesis, OPCs are activated and become highly proliferative after demyelinating injury.^110^, ^111^ In addition, it has been shown that Olig2^+^ and NG2^+^Olig2^+^ cells increase dramatically in the perihematoma region after ICH^4^ and that this increase is not due to migration of oligodendrocyte‐lineage cells from the subventricular zone (SVZ)—the site of oligodendrogenesis.^4^, ^112^ Together, these findings strongly indicate that ICH induces OPC proliferation.

### 3.3 OPC differentiation after ICH

After injury, OPCs proliferate and differentiate into mature‐OLs, which remyelinate damaged axons through a series of processes, including contact with demyelinated axons, myelin membrane production, and ensheathment of target axons.^111^ It should be noted that our understanding of the remyelination process mainly comes from demyelinating diseases, such as multiple sclerosis. How ICH affects OPC differentiation and remyelination, however, remains largely unknown. One recent study showed that the density of mature‐OLs increased and peaked at day 7 after ICH at the perihematoma region.^4^ Although this finding suggests that OPCs are able to differentiate into mature‐OLs after ICH, it remains unclear whether these mature‐OLs are able to remyelinate damaged axons at the functional level. Thus, future studies should focus on characterizing OPC differentiation and remyelination after ICH. Understanding the time course of these changes will substantially deepen our knowledge in ICH pathogenesis and promote the development of effective treatments for ICH.

## 4 CROSSTALK BETWEEN OLIGODENDROCYTES AND OTHER CELLS

Oligodendrocytes function to myelinate axons during development and remyelinate damaged axons after injury. These processes are tightly regulated by a variety of signaling pathways from different cell types. It is important to understand the interactions between oligodendrocytes and other brain cells, taking into account the crucial functions of oligodendrocyte‐lineage cells in white matter damage repair.^113^ Here, we summarize crosstalk between oligodendrocytes and other cells in the brain. For a summary of the factors from other brain cells and their functions in oligodendrocyte biology, please refer to Table 3.

**Table 3:** Factors from other brain cells and their functions in oligodendrocyte biology

Cell type	Factors	Function
Neurons	Neuregulin‐1	OPC proliferation
	CNTF	OPC proliferation, differentiation, myelination, OL apoptosis
Astrocytes	pStat3	OPC activation, myelination
	TNFα‐TNFR2	OPC proliferation, differentiation, remyelination, OL maturation
	BDNF	Oligodendrogenesis
Endothelial cells	FGF	OPC proliferation
	BDNF	OPC proliferation
Microglia	Activin‐A	OL differentiation, remyelination

### 4.1 Crosstalk between oligodendrocytes and neurons

As the target of myelination/remyelination, neurons are well‐positioned to talk with oligodendrocytes. Although not absolutely required for myelination,^75^ neurons have been shown to actively regulate myelination via neuregulin‐1. For example, glial growth factor, the soluble neuregulin‐1 isoform, has been found to promote OPC proliferation.^114^ In addition, neuronal expression of neuregulin increases significantly in the penumbra at day 3 after permanent middle cerebral artery occlusion (MCAO).^115^ Furthermore, neuregulin‐1 has been found to inhibit cortical damage, apoptosis, and inflammatory responses in an MCAO model.^116^ Based on these results, we hypothesize that neuronal neuregulin‐1 induces OPC proliferation and regulates injury pathology/outcome in ICH.

Another neuronal factor that affects oligodendrocyte function is CNTF (ciliary neurotrophic factor).^117^ A strong promyelinating effect of CNTF has been proposed based on the enzymatic index of myelination.^118^ Using transgenic mice without CNTF, it has been shown that CNTF enhances OPC proliferation, reduces oligodendrocyte apoptosis, and protects myelin integrity and function,^119^ again suggesting a promyelinating effect of CNTF. It is thus speculated that CNTF induces oligodendrocyte differentiation and promotes remyelination after ICH. Future studies should focus on investigating how exactly neurons crosstalk with oligodendrocytes and how this interaction affects disease progression.

### 4.2 Crosstalk between oligodendrocytes and astrocytes

Astrocyte‐oligodendrocyte crosstalk has been well documented. Accumulating evidence suggests that astrocytes actively regulate OPC differentiation and myelination via secreted growth factors and chemokines.^120^, ^121^ First, oligodendrocyte‐mediated remyelination occurs in the areas with astrocytes.^122^ Next, OPC activation and maturation as well as myelin formation are significantly attenuated in transgenic mice lacking phosphorylated STAT3 (signal transducer and activator of transcription 3) specifically in astrocytes (GFAP‐STAT3‐CKO mice),^122^ suggesting that pStat3 signaling in astrocytes contributes to OPC activation and subsequent myelination. Third, it has been shown that TNFα (tumor necrosis factor α)‐TNFR2 signaling in astrocytes induces the expression of CXCL12 (C‐X‐C motif chemokine 12), which regulates OPC proliferation, differentiation, and remyelination by binding to its receptor CXCR4.^121^ In addition, there is also evidence showing that TNFR2 signaling promotes oligodendrocyte maturation via LIF (leukemia inhibitory factor).^123^ Furthermore, astrocyte‐derived BDNF has also been demonstrated to positively regulate oligodendrogenesis after white matter damage.^124^ Although none of these studies were performed in ICH condition, it is logical to hypothesize astrocyte‐oligodendrocyte crosstalk induces OPC differentiation and remyelination after ICH in a similar way. This hypothesis will be tested in future studies.

### 4.3 Crosstalk between oligodendrocytes and endothelial cells

Endothelium‐oligodendrocyte interaction has been reported in previous studies. For example, it has been shown that OPCs make physical contact with brain endothelial cells and migrate along the vasculature during development.^17^ This correlation between oligodendrocytes and brain endothelial cells has also been found in pathological conditions. It has been reported that oligodendrocytes facilitate angiogenesis partially via upregulating matrix metalloproteinase‐9 (MMP9) after white matter injury.^125^ Similarly, vessel density partially correlates with the number of Olig2^+^ cells and oligodendrocytes in an ischemic stroke model,^126^ and strong angiogenic activity is found in the SVZ, the main neural stem cell niche that produces OPCs after demyelinating injury.^127^ In addition, OPCs have been shown to enhance the blood‐brain barrier integrity by secreting TGF‐β1 and upregulating tight junction proteins.^128^ Furthermore, cerebral endothelial cells have been speculated to stimulate OPC proliferation by secreting FGF and BDNF via Src and AKT signaling pathways.^24^ It is worth noting that the above‐mentioned studies were not done in ICH condition. Future studies should investigate how exactly endothelial cells and oligodendrocytes talk to each other after ICH.

### 4.4 Crosstalk between oligodendrocytes and microglia

Microglia are one of the first cell types activated after ICH. Activated microglia are classified into two main states: proinflammatory M1 state and antiinflammatory M2 state.^129^ These two states have different dynamics after ICH. For example, M1 polarization peaks as early as 4 hours after ICH, whereas M2 polarization peaks 24 hours after ICH.^130^, ^131^ Although how exactly microglia communicate with oligodendrocytes remains unknown, it is believed that microglia‐derived cytokines are able to regulate oligodendrocyte differentiation and myelination. For example, M2 microglia have been shown to promote oligodendrocyte differentiation and remyelination via activin‐A in demyelinating injury.^132^ Further studies should address whether and to what extent M2 microglia‐derived activin‐A contributes to oligodendrocyte differentiation and remyelination after ICH.

## 5 OLIGODENDROCYTE‐BASED THERAPIES

Due to the important functions of oligodendrocytes in myelination/remyelination, oligodendrocyte‐based therapies have attracted a lot of attention. It has been shown that OPC transplantation following spinal cord injury significantly improves the percentage of myelinated axons^133^ and stimulates functional recovery.^134^, ^135^ In addition, OPC transplantation has also been shown to induce myelin sheath formation, stimulate neural stem cell proliferation, facilitate spatial learning and memory recovery, promote BDNF and Bcl‐2 expression, and inhibit neuronal apoptosis in a rat model of periventricular leukomalacia.^136^ Based on these findings, it is speculated that oligodendrocytes may play a neuroprotective role in ICH by promoting remyelination and aiding in the repair process after injury. This hypothesis will be tested in future research. If a beneficial role of oligodendrocyte‐lineage cells is observed in ICH, these cells may be targeted to develop novel therapies for ICH. Specifically, minimizing oligodendrocyte death/enhancing oligodendrocyte survival and promoting oligodendrocyte differentiation/maturation should be able to improve ICH outcome. A thorough understanding of the biology of oligodendrocyte‐lineage cells and their functions in ICH will open doors for novel and effective treatments for this devastating disease.

## CONFLICT OF INTEREST

The authors declare no conflicts of interest.
